# Supplementary material for: 3D-printed self-healing hydrogels via Digital Light Processing
Source: Nat Commun. 2021 Apr 28;12:2462. doi: 10.1038/s41467-021-22802-z (PMC8080574; doi:10.1038/s41467-021-22802-z)
Supplement: Supplementary file 1 — Supplementary Information [file 41467_2021_22802_MOESM1_ESM.pdf]

Supplementary Information for 3D-printed self-healing hydrogels via Digital Light Processing

Matteo Caprioli[1,2], Ignazio Roppolo[1], Annalisa Chiappone[1], Liraz Larush[2], Candido Fabrizio Pirri[1,3] and Shlomo Magdassi[3]

[a] Department of Applied Science and Technology, Politecnico di Torino, Corso Duca degli Abruzzi 24, 10129 – Turin, Italy. [b] Casali Center for Applied Chemistry, Institute of Chemistry, The Hebrew University of Jerusalem, Edmond J. Safra Campus – Givat Ram, 9090145 - Jerusalem, Israel. [c] Istituto Italiano di Tecnologia, Center for Sustainable Future Technologies, Via Livorno 60, 10144 - Turin, Italy.

Correspondence and requests for materials should be addressed to I.R. (Ignazio.roppolo@polito.it) and S.M. (magdassi@mail.huji.ac.il)

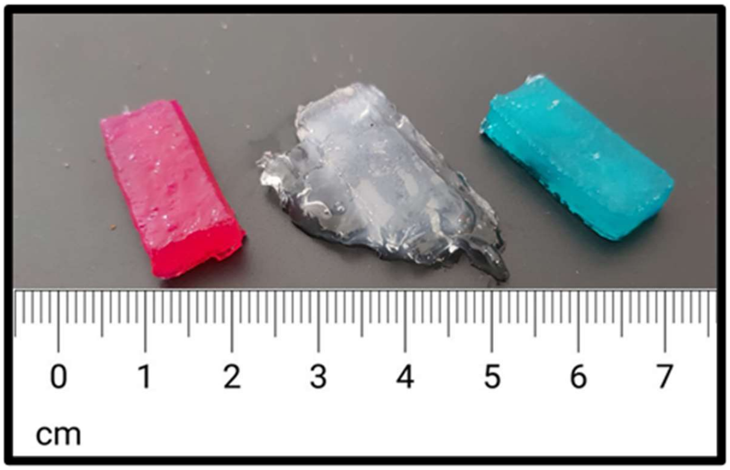

Supplementary Figure 1. Effect of the addition of the dye on the lateral resolution: Cuboid-shaped samples (25 mm x 10 mm x 5 mm) printed with formulations containing two different dyes, methyl red (left) and brilliant green (right), compared to a sample printed without adding a dye (center).

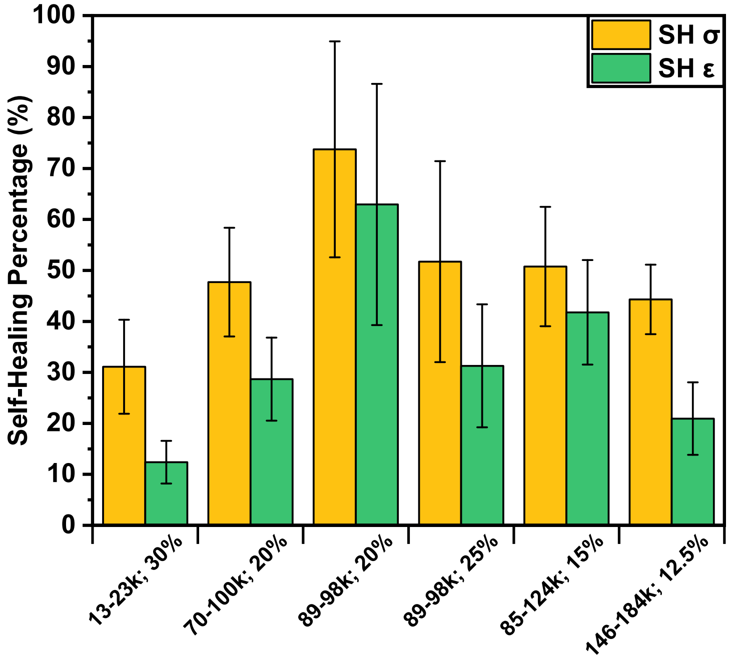

Supplementary Figure 2. Comparison of the healing efficiencies estimated from tensile strength at break (SHσ) and elongation at break (SHε) for formulations containing PVA with different molecular weights (MW) at the highest concentration in water which is still suitable for printing. Error bars represent standard deviation, n = 5 independent replicates.

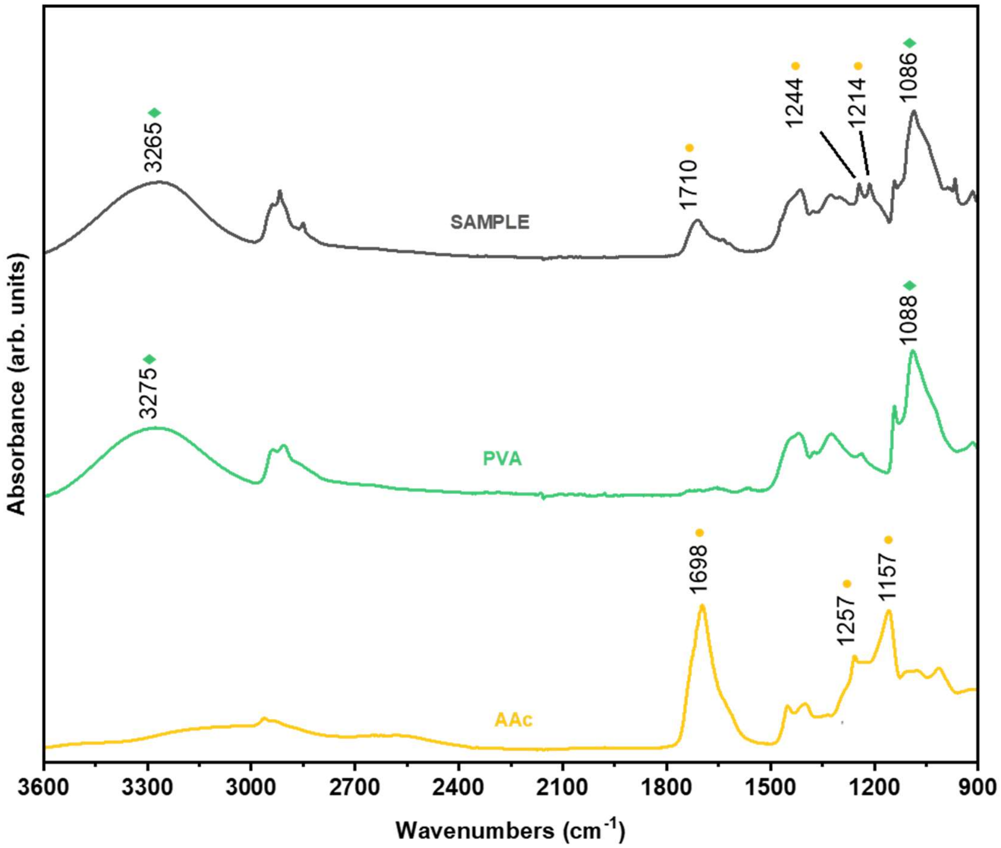

Supplementary Figure 3. ATR-IR spectra of Acrylic Acid (AAc), Poly (vinyl alcohol) (PVA) and of a dried sample. The assignment of the characteristic peaks in the different spectra is reported in Supplementary Table 3.

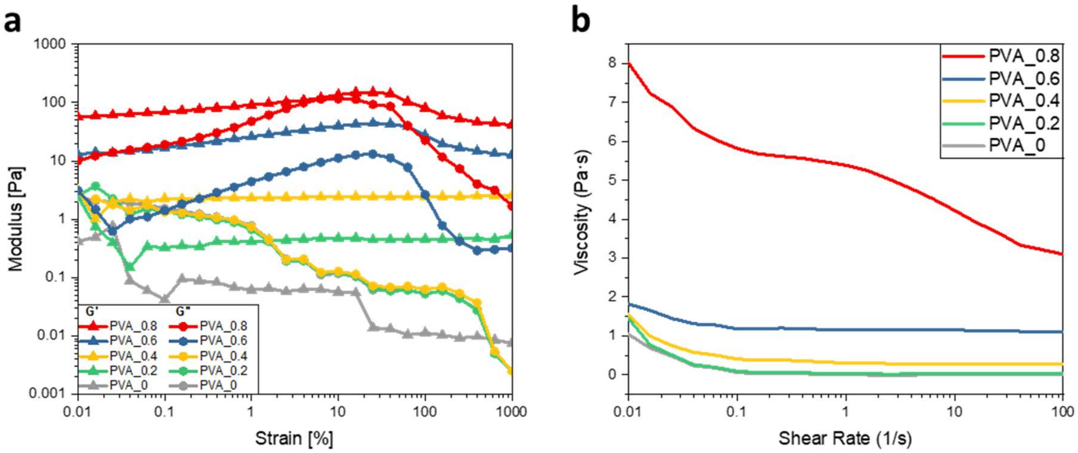

Supplementary Figure 4. a) Storage G′ and loss G″ moduli of formulations with increasing PVA contents in strain amplitude sweep test at constant frequency (10Hz). b) Viscosities of formulations with increasing PVA contents under continuous shear rate sweep.

As expected, the rheological behavior of the inks was strongly related to the amount of PVA, with an increase in both storage (G′) and loss (G″) shear moduli with an increase of the polymer percentage. Similarly, viscosity at room temperature moderately increased when increasing PVA. Surprisingly, PVA_0.8 formulation (the number indicates the weight ratio between PVA and AAc) showed a sharp increase in viscosity, determining an upper limit in the maximum content of PVA attainable due to limitations in the mixing step. Furthermore, viscosity is a very important limiting parameter for vat 3D printing, and its effects had to be addressed during the process.

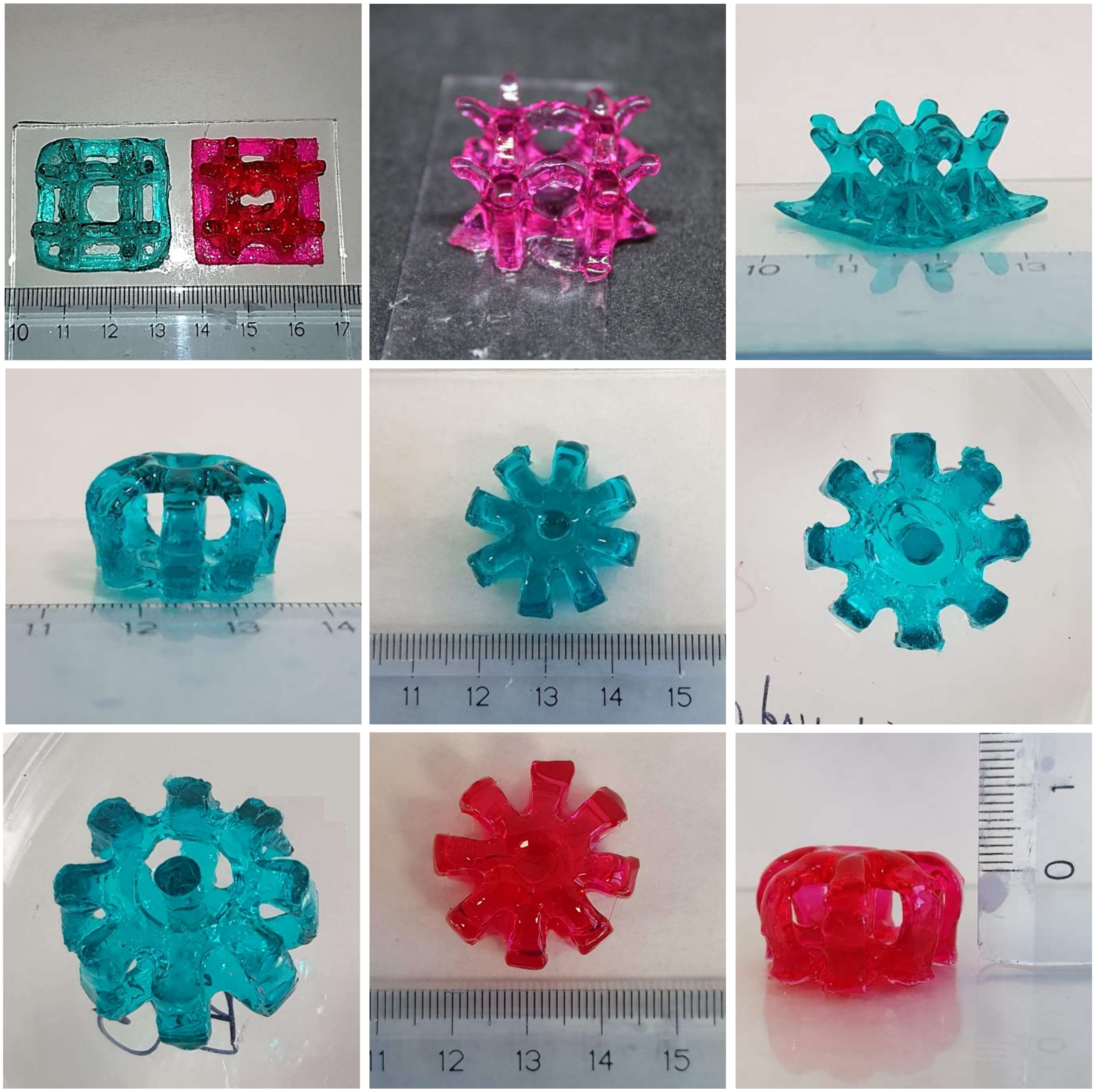

Supplementary Figure 5. Pictures from various angles of body-centered cubic lattice-like (first row) and axisymmetric structure with central pillar (second and third row).

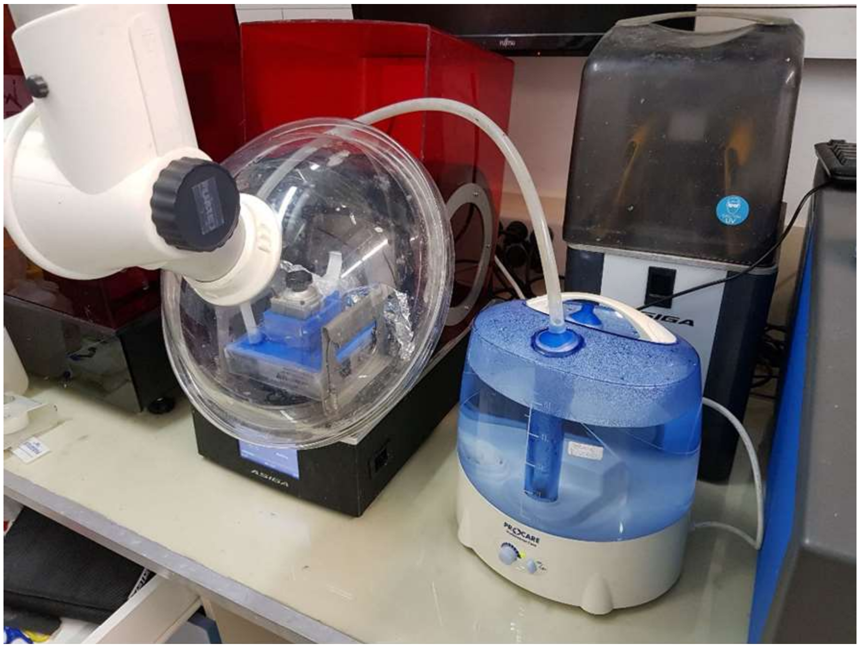

Supplementary Figure 6. Aerosol printing system configuration.

The evaporation of the water causes a surface solidification of the ink. This solid film hinders the printed object from reaching the bottom of the vat and immersing in the liquid resin, impeding the completion of the printing.

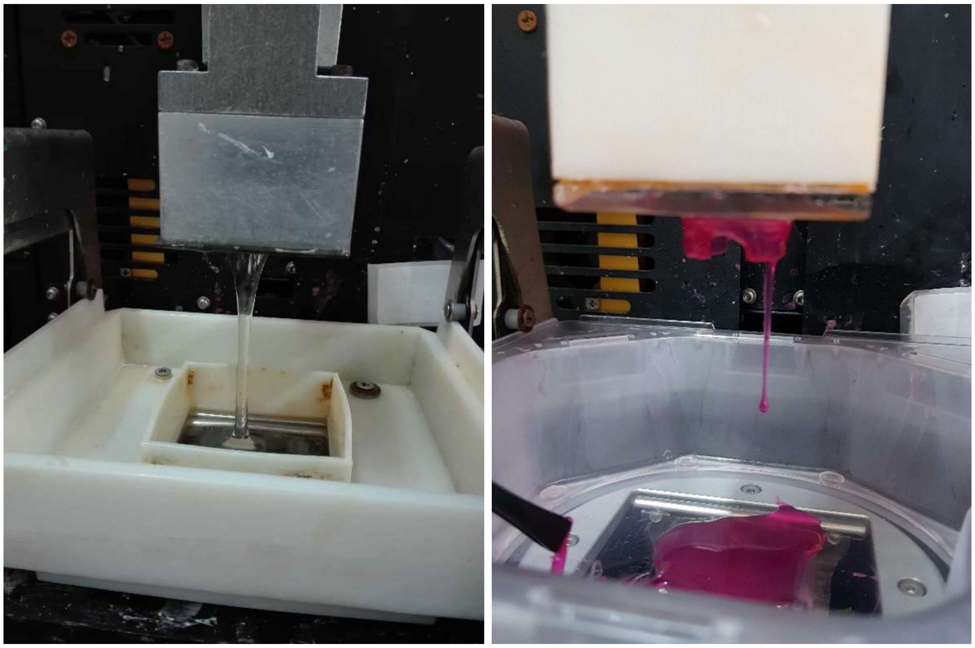

Supplementary Figure 7. Comparison of the rheological behavior of the PVA_0.8 during printing before (left) and after the addition of the dye (right).

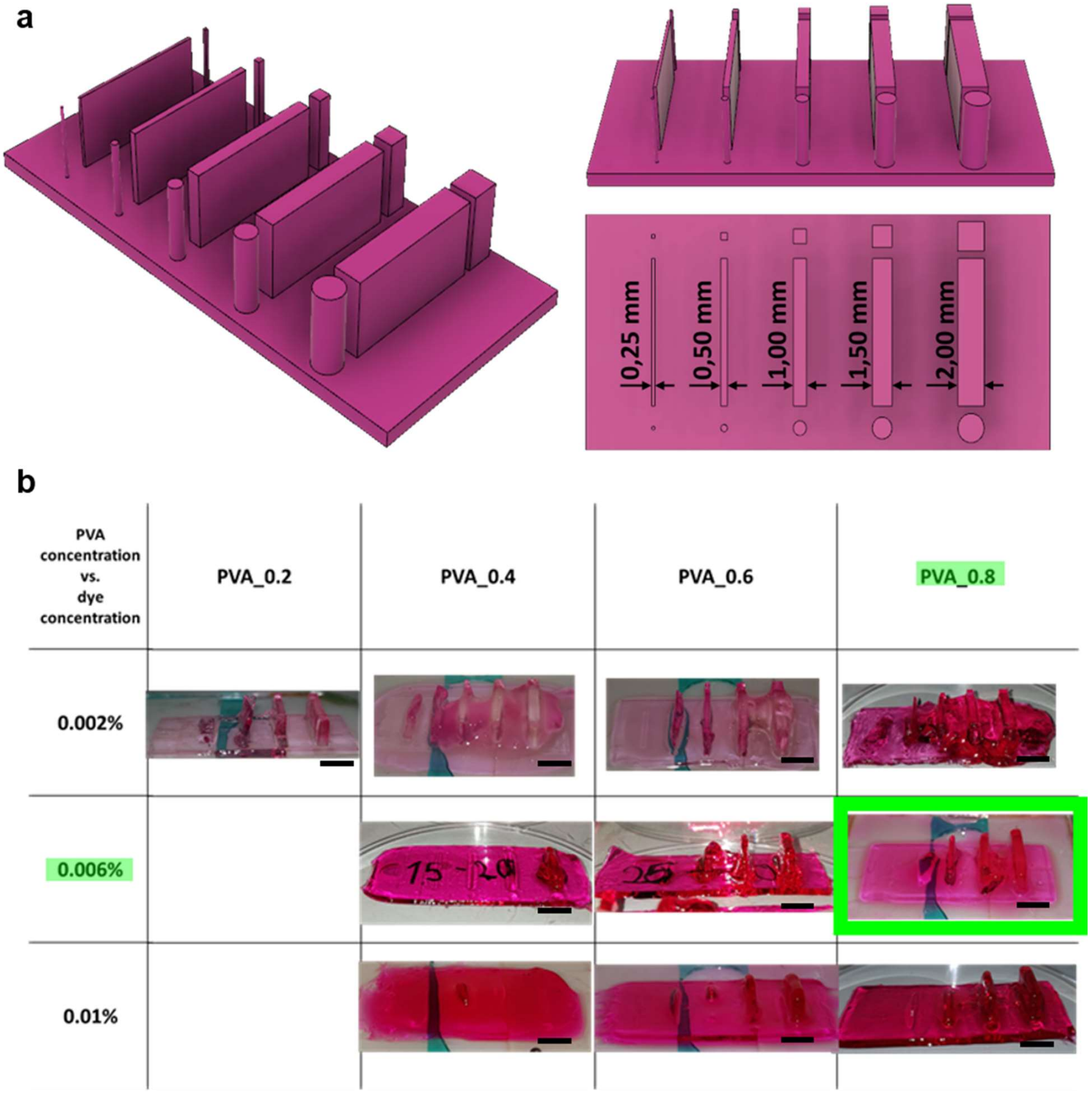

Supplementary Figure 8. a) Benchmark used to determine the finest feature possible to print. b) Table comparing the effects of the concentration of the PVA versus the concentration of the dye in the formulation on printed benchmarks. The walls on the samples have a height of 5 mm and a thickness of, from right to left, 2 mm, 1.5 mm, 1 mm, 0.5 mm, 0.25 mm (scale bar 5 mm).

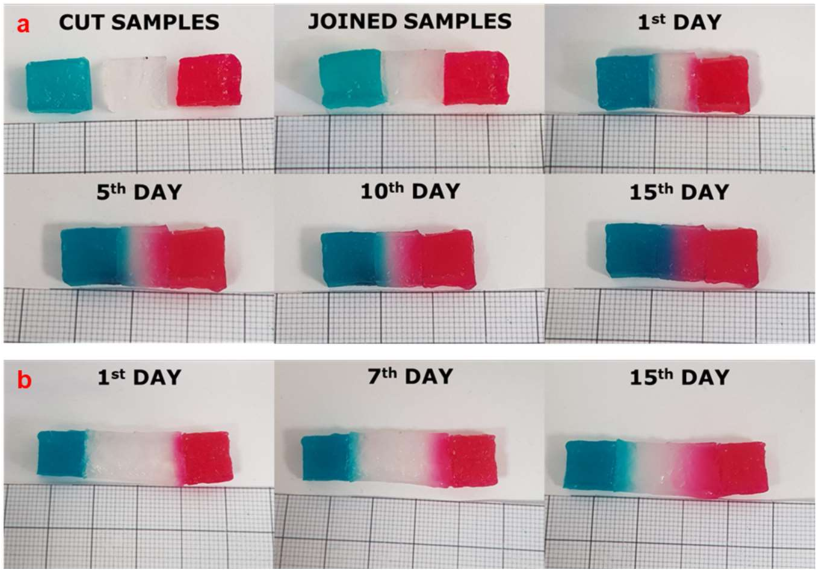

Supplementary Figure 9. Diffusion experiments of different dyes in a similar matrix. a) Similarly-sized cuboid in contact for several days to show diffusion an mixing of the dyes. b) Cuboids with longer non-colored central part to show differences in the diffusion speed.

Cuboid-shaped samples (25 mm x 10 mm x 5 mm) were printed with formulations containing the two different dyes and without the dye. The colored samples were cut in half attached two differently colored halves to the undyed sample, from where the two ends were cut to expose fresh surfaces. The samples were stored in a sealed vessel to minimize water evaporation. As it can be seen, methyl red migrates more rapidly than brilliant green in the central part. The two molecules show different diffusivity in the hydrogel, probably due to their different hindrance, nature, and affinity with the matrix, so they need a different time to diffuse along the same length. After 24 hours, methyl red shows visible diffusion in the central part, while brilliant green does not. After 15 days the diffusion gradients are in contact, with a purple color like the samples shown in the manuscript. The prolongation of the diffusion path helps to show the differences in the diffusion kinetic of the two dyes. It can be concluded that the diffusion of methyl red is faster and tends to hinder the diffusion of the brilliant green.

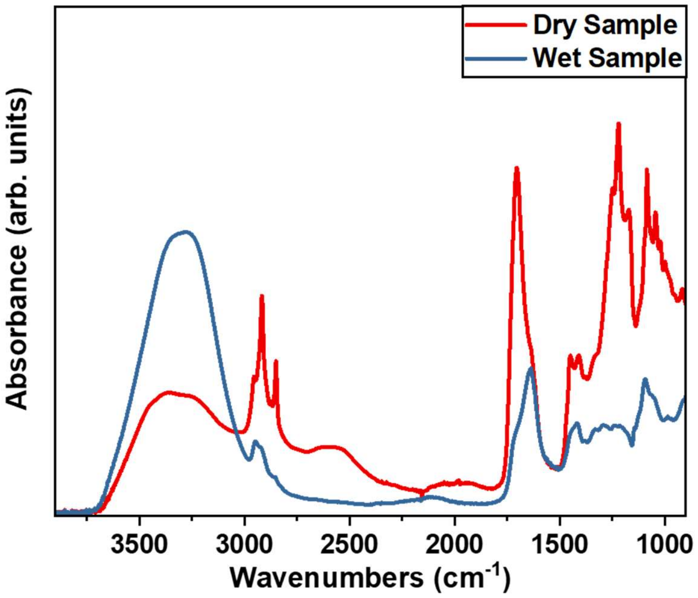

Supplementary Figure 10. ATR-IR spectra comparison of an as-printed sample (wet sample) and a dried sample (dry sample). It can be noted that the bands of hydroxyl groups of the PVA in the wet sample are hidden by the dominant bands of water (broad peak around 3200 cm^{-1}).

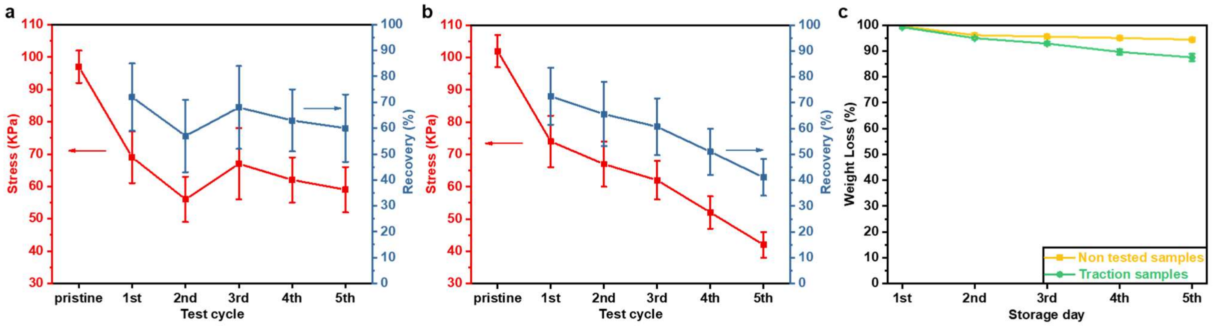

Supplementary Figure 11. a) Tensile strength and recovery percentage of samples after five separation-healing cycles, with samples stored in humid sealed environment for 24 hours while healing for every cycle. Error bars represent standard deviation, n = 6 independent replicates. b) Tensile strength and recovery percentage of samples after five separation-healing cycles, with samples stored in a sealed vessel with no controlled humidity for 24 hours while healing for every cycle. Error bars represent standard deviation, n = 6 independent replicates. c) Comparison of weight loss during several days between samples only stored in closed and sealed vials and samples subjected to manipulation for tensile testing. Error bars represent standard deviation, n = 5 independent replicates.

Repetitive self-healing properties were evaluated on 3D printed dumbbell-shaped specimens tested in tensile tests. the samples were tested for five consecutive days every 24 hours within each test, rejoining the samples after rupture and keeping them in a sealed environment with and without controlled humidity. As can be seen, the healing in a humid environment shows an average value of about 65% with certain repeatability that falls in the error range. The storage with uncontrolled humidity resulted in a restoration that took place every time with a lower efficiency, while the first healing process resulted as efficient as the humidly stored samples. This behavior can be explained considering the minor loss of weight that we measured during the time. This weight loss, unfortunately, could not be avoided because of water evaporation during handling and testing. The variability in the results can be mostly attributed to the manual reattachment of the samples, which could not always be rejoined in the same exact way. However, we performed the tests on multiple specimens to reduce the experimental uncertainty.

Supplementary Table 1. Characteristics comparison of extrusion printing and vat photopolymerization

Process	Material	Viscosity [1,2]	Material Deposition [3]	Model Surface [4]
Material Extrusion (FDM, FFF, DIW)	Viscous Liquids<br>Thermosoftening polymers	10^4-10^7 mPa·s	Continuous line	Very rough
Vat Photopolymerization (SLA, DLP, CLIP)	Liquid Photocurable resins	10^0-10^3 mPa·s	SLA – Line rastering<br>DLP Layer exposure	Smooth

Process	X-Y Resolution [5]	Z Resolution [4]	Fabrication Speed [6]	RTM Ratio* [7]
Material Extrusion (FDM, FFF, DIW)	100-200 µm	100-200 µm	Slow (µm/s)	0.5-1 10^{-3} m^2/min
Vat Photopolymerization (SLA, DLP, CLIP)	SLA 1-10 µm<br>DLP 20-200 µm	> 1 µm	SLA - Medium (mm^2/s)<br>DLP - Fast (mm^3/s)	0.5-2 10^{-3} m^2/min

*RTM Ratio: Resolution/time of manufacturing Ratio

Supplementary Table 2. Assignment of the characteristic peaks in the spectra reported in Supplementary Figure 3.

Peak Assignment	PVA	AAc	SAMPLE
PVA OH stretching	3275 cm^{-1}	—	3265 cm^{-1}
AAc C=O stretching	—	1698 cm^{-1}	1710 cm^{-1}
AAc C-OH stretching	—	1257 cm^{-1}	1244 cm^{-1}
AAc C-OH bending	—	1157 cm^{-1}	1214 cm^{-1}
PVA OH bending	1088 cm^{-1}	—	1086 cm^{-1}

Supplementary Table 3. Formulations weight composition.

BATCH	AAc	PVA	DIW	PEGDA	PI	DYE
PVA 0	23.67%	0%	75.73%	0.24%	0.36%	0.007%
PVA 0.2	22.60%	4.52%	72.31%	0.23%	0.34%	0.007%
PVA 0.4	21.62%	8.65%	69.18%	0.22%	0.32%	0.006%
PVA 0.6	20.72%	12.43%	66.32%	0.21%	0.31%	0.006%
PVA 0.8	19.90%	15.92%	63.68%	0.20%	0.30%	0.006%

Supplementary Table 4. Apparent crosslinking density.

Formulation	Apparent crosslinking density (m^{-3})
PVA 0	4.6×10^{23}
PVA 0.2	1.0×10^{24}
PVA 0.4	1.5×10^{24}
PVA 0.6	2.6×10^{24}
PVA 0.8	4.0×10^{24}

Supplementary References

1. Kyle, S., Jessop, Z. M., Tarassoli, S. P., Al-Sabah, A. & Whitaker, I. S. Assessing printability of bioinks. in *3D Bioprinting for Reconstructive Surgery* 173–189 (Elsevier, 2018). doi:10.1016/B978-0-08-101103-4.00027-2
2. Mendes-Felipe, C., Oliveira, J., Etxebarria, I., Vilas-Vilela, J. L. & Lanceros-Mendez, S. State-of-the-Art and Future Challenges of UV Curable Polymer-Based Smart Materials for Printing Technologies. *Adv. Mater. Technol.* **4**, 1800618 (2019).
3. Lee, J. M. & Yeong, W. Y. Design and Printing Strategies in 3D Bioprinting of Cell-Hydrogels: A Review. *Adv. Healthc. Mater.* **5**, 2856–2865 (2016).
4. George, E., Liacouras, P., Rybicki, F. J. & Mitsouras, D. Measuring and Establishing the Accuracy and Reproducibility of 3D Printed Medical Models. *RadioGraphics* **37**, 1424–1450 (2017).
5. Ahangar, P., Cooke, M. E., Weber, M. H. & Rosenzweig, D. H. Current Biomedical Applications of 3D Printing and Additive Manufacturing. *Appl. Sci.* **9**, 1713 (2019).
6. Zhang, J., Hu, Q., Wang, S., Tao, J. & Gou, M. Digital Light Processing Based Three-dimensional Printing for Medical Applications. *Int. J. Bioprinting* **6**, 1 (2019).
7. Moroni, L. *et al.* Biofabrication: A Guide to Technology and Terminology. *Trends Biotechnol.* **36**, 384–402 (2018).
