# Supplementary material for: 3D-printed self-healing hydrogels via Digital Light Processing
Source: Nat Commun. 2021 Apr 28;12:2462. doi: 10.1038/s41467-021-22802-z (PMC8080574; doi:10.1038/s41467-021-22802-z)
Supplement: Supplementary file 3 — Description of Additional Supplementary Files [file 41467_2021_22802_MOESM3_ESM.pdf]

Description of Additional Supplementary Files

File Name: Supplementary Movie 1
Description: Stretching of a 3D-printed healed object 12h after re-joining

File Name: Supplementary Movie 2
Description: : Cut and non-cut surfaces adhesion and healing comparison
